# Supplementary material for: The Molecular Chaperone Mechanism of the C-Terminal Domain of Large-Size Subunit Catalases
Source: Antioxidants (Basel). 2023 Mar 30;12(4):839. doi: 10.3390/antiox12040839 (PMC10135305; doi:10.3390/antiox12040839)
Supplement: Supplementary file 1 [file antioxidants-12-00839-s001.zip › antioxidants-2235525-supplementary.pdf]

Supplementary figures

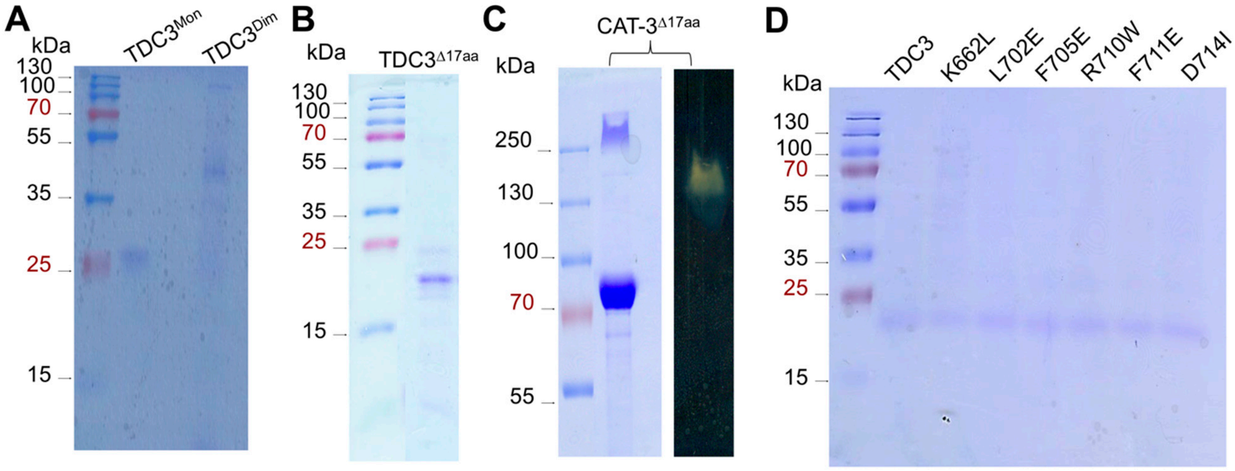

Figure S1. SDS-PAGE of TDC3 and CAT-3 variants**.** A) PAGE of TDC3^{Q652C} in monomer and dimer conformation (15 % of acrylamide). B) PAGE of TDC3$^{\Delta 17aa}$ (15 % of acrylamide). B) PAGE of CAT-3$^{\Delta 17aa}$ and in gel catalase activity (8 % of acrylamide); zymogram was run under non-denaturing conditions. C) PAGE of TDC3 single amino acid substitutions (15 % of acrylamide).

A

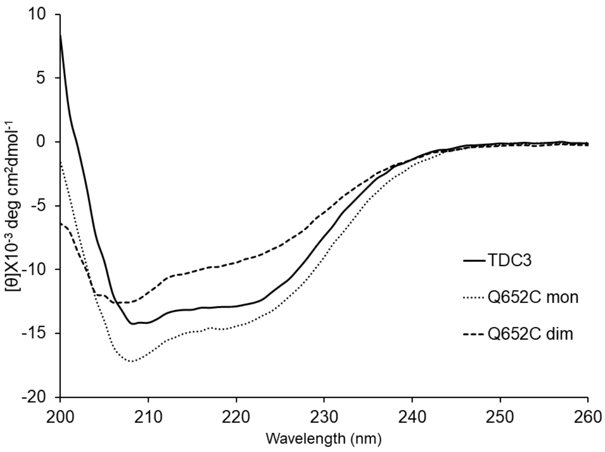

B

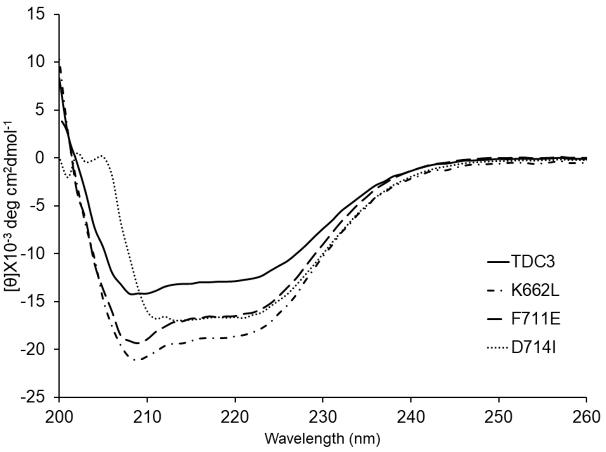

C Estimation of structural content by two servers

	BestSel (%)			K2D2 (%)		
	α helix	β strand	Others	α helix	β strand	Others
TDC3	88.4	9.2	2.5	84.27	1.24	14.49
Q652C mon	65.7	10.7	23.6	84.27	1.24	14.49
Q652C dim	50	13.9	36.1	75.57	1.72	22.71
K662L	13.5	0	86.5	84.27	1.24	14.49
F711E	51.5	17.1	31.1	84.27	1.24	14.49
D714I	33.4	66.7	0	84.27	1.24	14.49

Figure S2. CD of the TDC3 and estimation of structural content. A) Near UV spectra of the TDC3 monomer and dimer. The TDC3, the TDC3^{Q652C} as monomer or as dimer. B) CD of the three TDC3 mutant variants. C) Estimation of structural content using two servers. All TDC3 variants have a similar spectrum with the exception of the TDC3^{D714I} which apparently has more alpha helices and less unstructured regions.

A

TDC3 Primary sequence:

TLRVGVLSTTKGGSLDKAKALKEQLEKDGLKVTVIAEYLASGVDQTYSAADATAFDAVVV

AEGAERVFSGKGAMSPLFPAGRPSQILTDGYRWGKPVAAVGSAKKALQSIGVEEKEAGVY

AGAQDEVIKGVEEGLKVFKFLERFAVDGDDEE

B

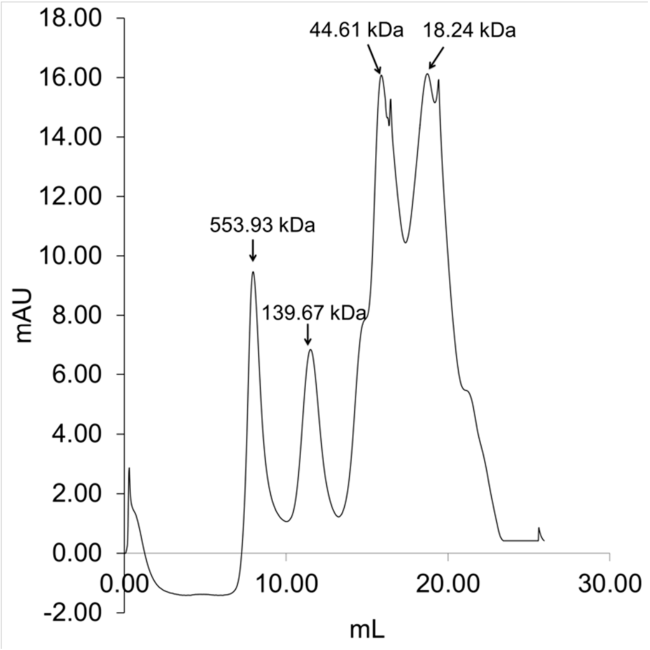

Figure S3. Primary sequence and molecular mass of TDC3. A) TDC primary sequence. B) Molecular mass of TDC3 determined by gel filtration chromatography. TDC3, 1mg/ml, was injected on a calibrated Superdex 75 HR 10/300 column. The solid line is the elution profile of TDC3.

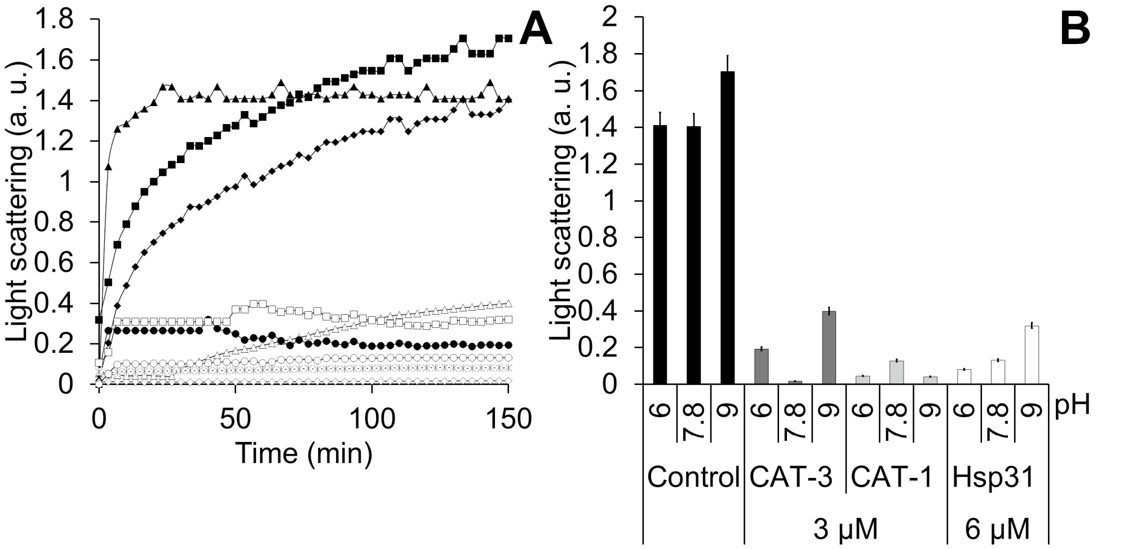

Figure S4. Unfolding activity of LSCs at various pH. A) LSCs effect on ADH heat denaturation (45°C) at various pHs. Light scattering at 360 nm generated by denaturation of ADH when incubated at 45°C. Control with BSA (6 µM): at pH 6 (closed triangles), pH 7.8 (closed diamonds) and pH 9 (closed squares). In the presence of CAT-3 (3 µM) at pH 6 (close circles), pH 7.8 (open diamonds) and pH 9 (open triangles). In the presence Hsp31 (6 µM) at pH 6 (open circles), at pH 7.8 (asterisks) and pH 9 (open squares). B) Average of the final light scattering value from three independent experiments. Buffers used: 50 mM sodium phosphate at pH 6, 50 mM Na/K phosphate at pH 7.8, and 50 mM sodium borate at pH 9.

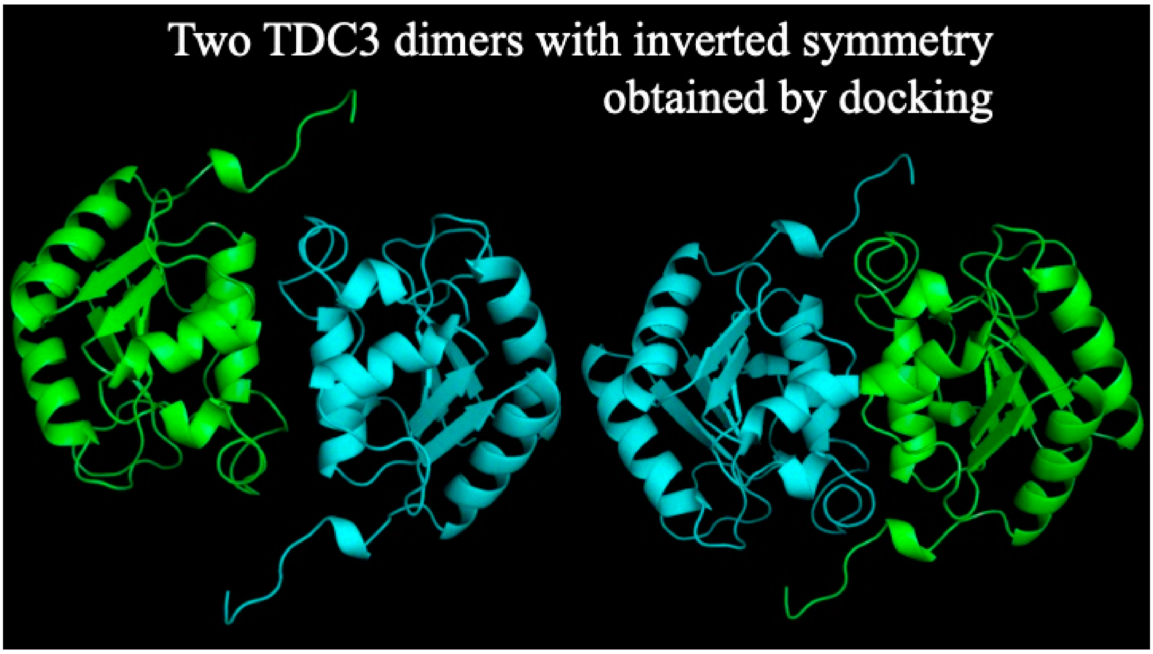

Figure S5. Formation by docking of TDC3 dimers with inverted symmetry which are similar to the dimers in CAT-3. We used the ClusPro server with standard parameters.
